# Gabapentinoids‐duloxetine combination therapy for chronic pain: A mechanism oriented rational to bridge theoretical knowledge and real life setting

**DOI:** 10.1002/bcp.70689

**Published:** 2026-07-24

**Authors:** Stefania Nobili, Maurizio Evangelista, Elena Lucarini, Elena G. Giugliano, Carla Ghelardini, Laura Micheli, Lorenzo Di Cesare Mannelli

**Affiliations:** ^1^ Department of Neuroscience, Psychology, Drug Research and Child Health University of Florence Florence Italy; ^2^ Institute of Anesthesia, Resuscitation and Pain Therapy Catholic University of Sacred Heart Rome Italy; ^3^ Department of Clinical and Experimental Medicine (DIMED) University of Messina Messina Italy

**Keywords:** antidepressants, antiepileptics, combination therapy, duloxetine, gabapentinoids, neuropathic pain

## Abstract

Chronic pain represents a complex debilitating condition that extends beyond the protective function of physiological pain, often persisting as an independent disease entity. Chronic primary and secondary pain syndromes reflect a multifaceted continuum involving nociceptive, neuropathic and nociplastic mechanisms. The maladaptive plasticity of the peripheral and central nervous system (encompassing the ascending and descending pain pathways) sustains hypersensitivity and correlates with comorbid alterations in mood, cognition, sleep and fatigue, underpinned by functional reorganization of brain networks. In this scenario, traditional analgesics frequently demonstrate limited efficacy, while current guidelines recommend antiepileptic agents and antidepressants, particularly gabapentinoids and duloxetine, as first line pharmacological options. This review explores the mechanistic rationale and clinical evidence supporting the combined use of gabapentinoids and duloxetine in chronic pain management. These agents act on distinct yet complementary targets: gabapentinoids reduce excitatory neurotransmission via modulation of calcium channel activity, whereas duloxetine restores descending noradrenergic inhibition and alleviates comorbid symptoms. Clinical trial and meta‐analyses highlight their individual efficacy in diabetic peripheral neuropathy, postherpetic neuralgia and fibromyalgia. Among gabapentinoids, pregabalin exhibits a favourable pharmacokinetic profile that allows rapid titration and demonstrates effectiveness against anxiety‐related sleep disorders. Importantly, emerging evidence suggests that their combination may offer additional benefit, particularly in patients with residual pain despite monotherapy, though evidence remains limited to small, short‐term studies. This review provides proof of concept by bridging theoretical knowledge and real‐life clinical settings aiming to develop treatment protocols based on predominant pain mechanisms that can effectively control hypersensitivity and improve quality of life.

## 1 INTRODUCTION

Chronic pain lacks the positive characteristic of physiological pain to early inform about a dangerous situation. Without the alarm role, the persistence of pain becomes an invalidating symptom and, in some cases, a per se disease. Following the recent classification proposed by the International Association for the Study of Pain (IASP) for the International Classification of Diseases (ICD‐11), chronic primary pain syndromes can be conceived as a pathology because they cannot be more appropriately ascribed to other chronic conditions; primary pain includes, for example, fibromyalgia, nonspecific low‐back pain or chronic primary visceral pain. Chronic secondary pain syndromes exhibit crucial factors to be considered in differential diagnosis (e.g., chronic cancer‐related pain, chronic neuropathic pain, chronic posttraumatic and postsurgical pain and chronic secondary visceral pain).^1^

Chronic pain is a dynamic condition where the three components of pain, nociceptive (involving tissue or potential tissue damage, according to IASP definition), neuropathic (involving disease or injury affecting the nervous system) and nociplastic (with no clear evidence of tissue or nerve damage but persistent overactivation of the somatosensory system, it is related to an altered CNS elaboration of pain signalling), interact and fluctuate over.^1^, ^2^, ^3^, ^4^ Even if conditions like diabetic neuropathy exemplify paradigmatic instances of neuropathic pain and fibromyalgia and nonspecific low‐back pain are emblematic cases of nociplastic pain, the predominance of any particular category may shift based on several factors, making pathological chronic pain a continuum that requires individualized, adaptive treatment strategies.^5^, ^6^ Pain is maintained over time and amplify in intensity by a maladaptive plasticity of the peripheral and central nervous system (PNS and CNS) that drives sensory hypersensitivity.^7^ The frequent presence of a parallel cluster of symptoms, including sleep, affect, mood, cognition and energy/fatigue alterations hypersensitivity, suggests a shared CNS dysfuntion in chronic pain.^8^, ^9^ This shared CNS dysfunction may also contribute to the frequent association between chronic pain and psychiatric comorbidities, particularly anxiety and depression. In this context, chronic pain is increasingly recognized as a multidimensional disorder involving maladaptive alterations in emotional, motivational and stress‐related neural systems. Shared mechanisms including limbic dysfunction, altered central neurotransmission, neuroinflammatory processes and maladaptive stress responses are thought to contribute to pain chronification, affective symptom burden and impaired quality of life.^10^, ^11^, ^12^ Accordingly, functional imaging‐based studies report a derangement of the functional organization of the brain.^13^

Due to the described complex maladaptive response of the nervous system, the classical analgesic drugs are lesser effective against pathological pain.^6^ The current therapy mainly includes antiepileptics and antidepressants.^7^ Among these categories, gabapentinoid compounds and duloxetine emerged as particularly relevant according to international guidelines (e.g., National Institute for Health and Care Excellence [NICE] guidelines and the European Federation of Neurological Societies [EFNS]). In addition, according to a recent meta‐analysis, gabapentinoids and duloxetine received a strong recommendation for use as first‐line therapy in neuropathic pain.^14^ Based on the potential complementary effects of gabapentinoids and duloxetine in pain reduction (Figure 1), this narrative review aims to evaluate the role of their combination in the management of pathological pain of different origins. To this end, the pivotal clinical trials that led to the approval of these drugs for such conditions are presented as key evidence supporting the rationale for their combined use. The potential application of this combination in chemotherapy‐induced peripheral neuropathy is also discussed. Overall, this is the first review to specifically address this topic, providing an updated overview of this pharmacological approach. As a proof of concept, this review represents a bridge between theoretical knowledge and real‐life settings to develop treatment protocols based on predominant pain mechanisms.

**FIGURE 1:**
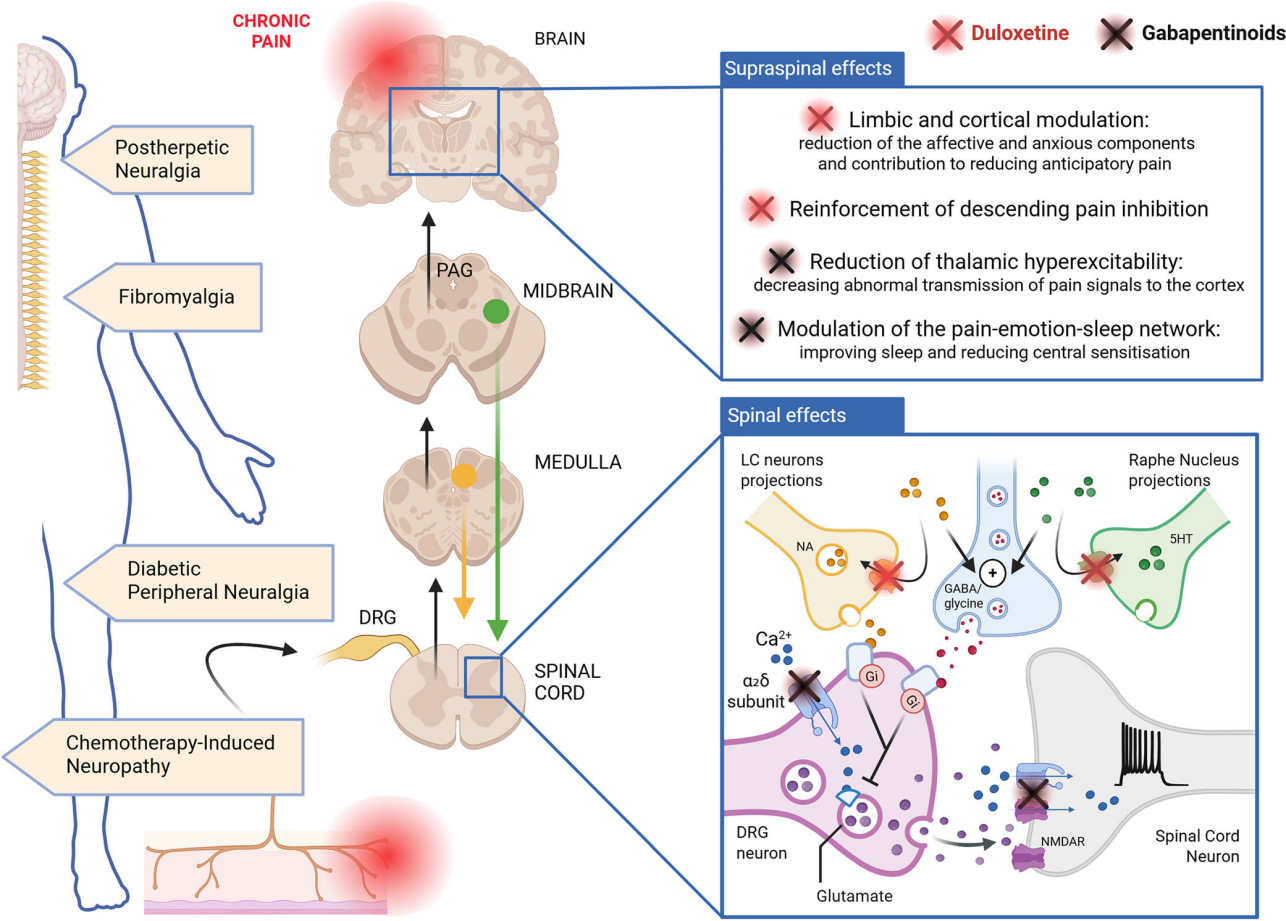
Complementary effects of gabapentinoids and duloxetine in chronic pain reduction.

### 1.1 Search strategy

This is a narrative review. The literature search was primarily conducted using PubMed, with additional screening of reference lists from relevant articles and international guidelines. The following keywords and combinations were used: ‘antiepileptics’, ‘antidepressants’, ‘duloxetine’, ‘gabapentinoids’, ‘nociceptive pain’, ‘neuropathic pain’ and ‘combination therapy’.

For the mechanistic and pathophysiological sections, no specific time restrictions were applied, in order to include seminal and more recent evidence regarding pain neurobiology, central sensitization, descending monoaminergic modulation and neuroimmune mechanisms involved in chronic pain.

For the clinical sections, the literature search was specifically oriented towards pivotal clinical trials, meta‐analyses and evidence supporting the regulatory approval of gabapentinoids and duloxetine for pain‐related indications. When approved indications were not available or evidence was limited, additional targeted PubMed searches were performed to identify relevant clinical studies evaluating these agents in specific pain conditions and combination therapy settings.

Articles were selected based on their relevance to the pharmacological mechanisms, clinical efficacy and translational implications of gabapentinoids and duloxetine in chronic pain management.

## 2 PAIN SIGNALLING

Regardless of whether the origin of pain is peripheral or central the evolution to a pathological pain involves neuronal hyperexcitability along the nociceptive neuraxis due to an imbalance of inhibitory and excitatory tone.^7^

### 2.1 Pain signalling under physiologically conditions

Nociceptive input arises from a specialized subset of peripheral nerve fibres called nociceptors which respond to thermal, mechanical or chemical stimuli.^15^ The cell bodies of these fibres are located in the dorsal root ganglia (DRG) for the body and in the trigeminal ganglion for the facial innervation. Their central projections terminate in the dorsal horn of the spinal cord, which is organized into anatomically and electrophysiologically distinct laminae. Within this structure, projection neurons in laminae I and V serve as the main output channels to supraspinal sites. These neurons contribute to several ascending tracts, most notably the spinothalamic pathway (involved in the sensory‐discriminative dimension of pain) and the spinoreticulothalamic pathway (which is associated with poorly localized or diffuse pain sensations), transmitting pain signals to the thalamus and brainstem, respectively.^15^ From there, nociceptive information is relayed to higher cortical regions, including the somatosensory cortex, as well as areas implicated in affective processing such as the anterior cingulate gyrus and insular cortex.^16^

Two principal ascending systems can be distinguished: the medial and lateral pain pathways, which are anatomically and functionally separated.^17^ The rostrodorsal anterior cingulate (rdACC) and anterior insular cortex are parts of the medial pain pathway. Functionally, the insula can be subdivided into three regions: the posterior insula, which processes sensory information including nociception; the anterior ventral insula, primarily engaged in emotional and social functions; and the dorsal anterior insula, which contributes to cognitive processing. The rdACC integrates cognitive, affective, somatosensory and autonomic inputs, making it a critical hub for linking negative emotions with the cognitive regulation of both acute and chronic pain. Within the medial pain pathway, the rdACC specifically encodes the unpleasant, emotional dimension of pain.^17^

Within this pathway, signal transmission is tightly regulated by a range of voltage‐gated ion channels. Sodium and potassium channels are fundamental for generating the action potentials that carry nociceptive signals to their synaptic targets. In parallel, voltage‐gated calcium channels are essential for neurotransmitter release at both central and peripheral nociceptor terminals.

Ascending pain pathways are counterbalanced by a descending inhibitory network that involves the rostral and pregenual anterior cingulate cortex (pgACC), the periaqueductal grey (PAG), parahippocampal area, hypothalamus and the rostral ventromedial brainstem.^18^ Huang et al.^19^ demonstrated in animal models that this inhibitory system originates in the amygdala (which establishes connections with pgACC), and further relayed to the PAG, brainstem and spinal cord.^19^ The central amygdala also integrated pain‐related signals from the spinal cord through inputs from the parabrachial nucleus.^20^ Neurochemically, this pathway relies on serotoninergic and noradrenergic transmission,^17^, ^19^ including serotonergic projections from the raphe nuclei and noradrenergic fibres descending from the locus coeruleus.^21^

Within the rostral ventromedial medulla (RVM), two principal classes of pain‐modulatory neurons have been identified: the on‐cells and the off‐cells. Off‐cells are activated by opioids and inhibited by noxious stimulation, exerting an inhibitory influence by engaging descending inhibitory circuits that dampen peripheral nociceptive input. In contrast, on‐cells display the opposite response pattern, triggering descending facilitation.^22^

### 2.2 Pain signalling under pathological conditions

The shift from physiological to pathological pain is driven by an imbalance between excitatory and inhibitory mechanisms, sustained by ion channel dysfunction, altered neurotransmitter release, glial activation, neuro–immune interactions and inflammatory processes. A complex maladaptive response of the nervous system underlies the main clinical manifestation, including spontaneous pain (such as burning sensations) and stimulus‐evoked pain in response to noxious (hyperalgesia) or non‐noxious (allodynia) stimuli. Neural plasticity contributes to this transition through both peripheral sensitization, involving primary sensory neurons in DRG and trigeminal ganglia,^15^, ^23^ and central sensitization of pain‐processing neurons within the spinal cord and brain.^24^, ^25^

Central sensitization is strongly influenced by neuroinflammation processes affecting both PNS and CNS.^26^ A hallmark of this condition is the activation of glial cells: in the periphery, Schwann cells and satellite glial cells in the dorsal root and trigeminal ganglia, and centrally, microglia, astrocytes and oligodendrocytes in the spinal cord and brain.^25^, ^27^ Persistent maladaptive pain arises from complex bidirectional interactions between neurons and glial cells, as well as from reciprocal crosstalk between neurons and immune cells such as T lymphocytes and macrophages^7^ as confirmed by the results of multiple positron emission tomography studies that described an increase of the 18 kDa translocator protein (TSPO), a putative neuroimmune marker, in patients with different pain condition.^26^

Neuroplastic alterations contribute to increased neuronal excitability through several mechanisms. These include post‐translational modifications, enhanced excitatory transmission mediated by glutamate receptors, particularly N‐methyl‐D‐aspartate (NMDA) and α‐amino‐3‐hydroxy‐5‐methyl‐4‐isoxazolepropionic acid (AMPA) receptors, and impaired clearance or buffering of glutamate by astrocytes, all of which promote neuronal hyperactivity within ascending pain pathways.^7^ Substance P (SP), co‐released with glutamate by unmyelinated peptidergic nociceptors, plays a central role in driving central sensitization. Calcitonin gene‐related peptide (CGRP), produced by small diameter sensory neurons, enhances the effects of SP and participates to central sensitization through activation of postsynaptic CGRP1 receptors, which stimulate protein kinase A (PKA) and PKC signalling.^28^ Growth factors, despite their neuroprotective properties, also has been shown to promote pain oversignaling; brain‐derived neurotrophic factor (BDNF) has long been implicated,^29^ and more recently, among others, vascular endothelial growth factor A.^30^

### 2.3 Calcium‐driven hyperexcitability and breakdown of inhibitory control in central sensitization

An increase in intracellular Ca^2+^ represents a pivotal trigger of nervous hyperactivity. Calcium influx occurs through several routes including ligand‐gated receptors as NMDA and AMPA,^28^ purinergic P2X7 receptors,^31^ as well as voltage‐gated calcium channels^32^ and transmembrane channels like pannexin 1.^31^ These influx mechanisms activate intracellular cascades involving phosphorylation, trafficking and transcriptional regulation. Ultimately, these processes alter neuronal excitability by enhancing inward currents, reducing outward currents and diminishing inhibitory signalling through impaired release or activity of GABA and glycine. Alongside mechanisms that enhance excitatory drive, pathological pain is sustained by a breakdown of descending modulatory control. Both endogenous opioid circuits and monoaminergic pathways, which normally maintain a balance between facilitation and inhibition of nociceptive transmission, become impaired.^22^, ^33^ A reduction in descending inhibitory tone, in the presence of decreased activity of inhibitory interneurons (arising from decreased synthesis of GABA and glycine or from the degeneration of interneurons),^34^ renders spinal neurons increasingly susceptible to excitatory inputs, even those originating from non‐nociceptive A‐fibres. This collapse of inhibitory mechanisms is widely recognized as a central driver in the initiation and persistence of central sensitization.^28^ At the spinal level, maladaptive plasticity is largely mediated by protein kinase C (PKC), which diminishes inhibitory transmission both locally and through descending control originating in the PAG. PKC reduces tonic GABAergic and glycinergic inhibition, thereby facilitating nociceptive signalling. Together with PKA, PKCcontributes to the activation of extracellular signal–regulated kinase (ERK), a process that requires the co‐activation of both kinases and is triggered by the central release of bradykinin.^28^ Furthermore, the increase of BDNFdownregulates the potassium–chloride cotransporter 2 (KCC2) in spinal neurons, thereby disrupting chloride homeostasis and weakening GABAergic inhibition.^35^

### 2.4 Maladaptive remodelling of descending monoaminergic pathways in the transition from acute to chronic pain

The disruption of inhibitory tone is further compounded by maladaptive changes in descending monoaminergic systems.^22^ The descending serotonergic pathways may exert either inhibition or facilitation, depending on the acute or chronic states of pain and the receptor subtypes involved. Serotonergic transmission may support descending inhibition, predominantly via 5‐HT_1_ receptors, as well as a shift towards facilitation mediated through 5‐HT_2_ and 5‐HT_3_ receptors.^22^, ^36^, ^37^ Experimental evidence shows that disruption of serotonergic projections from the RVM attenuates allodynia and hyperalgesia, confirming their facilitatory role in neuropathic pain.^38^ Moreover, chloride dysregulation due to impaired KCC2 function can further transform serotonergic input from inhibitory to excitatory, reinforcing maladaptive signalling.^39^

In contrast, the noradrenergic pathway retains predominantly inhibitory properties. Noradrenaline (NA) released from descending fibres suppresses nociceptive transmission by activating α_2_‐adrenergic receptors located presynaptically on primary afferents (reducing neurotransmitter release) and postsynaptically on dorsal horn neurons (enhancing hyperpolarization and decreasing excitability).^40^, ^41^ Stimulation of presynaptic α_2_‐adrenergic receptors suppresses voltage‐gated Ca^2+^ channel activity, thereby reducing the release of excitatory neurotransmitters such as glutamate and SP.^41^ In parallel, activation of postsynaptic α_2_‐adrenergic receptors on second‐order sensory neurons in the spinal cord induces hyperpolarization via inwardly rectifying potassium channels, leading to decreased neuronal excitability.^42^ Postsynaptic α_1_‐adrenergic receptors may also contribute to analgesia by promoting the release of inhibitory neurotransmitters, including GABA and glycine, from local interneurons.^40^

Although these mechanisms highlight the preponderantly antinociceptive role of spinal noradrenergic signalling, the noradrenergic system can also exert pronociceptive effects at supraspinal levels. In particular, direct modulation of brainstem pain circuits, such as the LC and the medullary dorsal reticular nucleus, may enhance pain transmission.^43^ Importantly, evidence indicates that in chronic pain states, the functional balance of the LC may shift from inhibition towards facilitation.^44^

Along the pain pathway, the noradrenergic pain modulatory system interacts with both different neurotransmitter systems (i.e., opioidergic and serotoninergic) and cellular partners (glial cells), providing an additional layer of modulation that contributes to the overall regulation of pain.^45^ At the spinal level, noradrenergic and serotonergic modulatory circuits act synergistically to enhance descending inhibition, thereby strengthening antinociceptive control.^46^, ^47^ In the early stages of pain, descending noradrenergic pathways exert a protective effect against mechanical and thermal hypersensitivity. This phenomenon is mediated by an increase in BDNFwithin the spinal dorsal horn, which, following nerve injury, fundamentally reshapes both the structure and function of noradrenergic circuits through activation of tropomyosin receptor kinase B. Upon activation, noradrenergic fibres sprout within the spinal dorsal horn at dermatomes surrounding the site of primary sensory input, enabling a more widespread release of NA. Furthermore, the function of α_2_‐adrenergic receptors on spinal cholinergic interneurons shifts from inhibitory to facilitatory. As a result, spinally released NA excites cholinergic interneurons, leading to acetylcholine release, which plays a critical role in the antihypersensitivity effects of NA after nerve injury.^42^ These molecular adaptations are accompanied by an upregulation and enhanced activity of both pre‐ and postsynaptic α_2_‐adrenergic receptors in the spinal dorsal horn.^48^

Later, a derangement in this signalling participates in the evolution from acute to persistent pain. Noradrenergic neurons of the LC become less responsive to noxious stimuli, largely due to dysfunction within the glutamatergic system that regulates NA release. Astroglial dysregulation of glutamate is particularly critical in this impairment.^49^ Additional alterations include upregulation of the NA transporter (NET) in the spinal cord of neuropathic models.^50^ Interestingly, although the number of spinal α_2_‐adrenergic receptors remains unchanged following spinal nerve ligation, their coupling efficiency with Gα subunits increases.^51^ These adaptive changes suggest that, following nerve injury, descending noradrenergic inhibitory tone is compromised, the improved efficiency of α_2_‐adrenergic receptor signalling may represent a compensatory mechanism that enhances the therapeutic efficacy of NA reuptake inhibitors.^51^ Beyond spinal circuits, supraspinal noradrenergic regions also undergo remodelling relevant to chronic pain. In long‐term neuropathic states, the LC exhibits increased expression of dopamine β‐hydroxylase, tyrosine hydroxylase, NET and α_2_‐adrenergic receptors. Although these changes may partially compensate for diminished spinal noradrenergic tone, they have also been implicated in the development of comorbidities such as anxiety and depression, commonly observed in chronic pain.^52^ In line with these mechanisms, the pain relieving efficacy of antidepressant like duloxetine is strongly attributed to its ability to engage descending noradrenergic pathways.^53^, ^54^ In addition to neurochemical changes, in the transition from acute to chronic pain, it has been reported structural (e.g., decrease in grey matter at the dorsolateral prefrontal cortex) and functional (enhanced connectivity in brain regions associated with pain dimension and decreased activation of the descending pain inhibitory system) changes^55^, ^56^ as well as a shift of brain activity from sensory circuits to emotion‐related circuits (e.g., mesolimbic).^57^, ^58^ Structural and functional brain abnormalities related to chronic pain are sensitive to effective treatments.^59^

## 3 GABAPENTINOIDS

Gabapentinoids (gabapentin, pregabalin and the more recently developed mirogabalin) were initially designed as lipophilic analogues of the inhibitory neurotransmitter GABA, with the aim of improving their penetration into the central nervous system while preserving GABA‐like pharmacological properties. Subsequent studies, however, demonstrated that their mechanism of action does not involve GABA receptors; gabapentinoids bind with high affinity to the auxiliary α_2_δ subunit of voltage‐sensitive calcium channels (VSCCs). Among these, the α_2_δ‐1 isoform plays the predominant role in mediating their pharmacological effects. This subunit is widely distributed in the nervous system, as well as in skeletal, cardiac and smooth muscle. Within the nervous system, α_2_δ‐1 is particularly abundant in DRGs, the spinal dorsal horn and multiple brain regions including the cerebral cortex, hippocampus and cerebellum. Expression of α_2_δ‐1 mRNA is enriched in areas involved in cortical processing, learning and memory, defensive behaviours, neuroendocrine control, autonomic regulation, arousal and sensory transmission.^60^

It is colocalized with glutamatergic excitatory neurons,^61^ and, in line with this distribution, α_2_δ‐1 expression is especially evident in VGluT2‐positive excitatory neurons of the spinal dorsal horn.^61^ Notably, elevated α_2_δ‐1 levels are closely linked to enhanced pain transmission: in animal models of neuropathic pain, DRG neurons show a marked upregulation of this subunit following peripheral nerve injury.^60^

By binding to the α_2_δ subunit of VSCC, gabapentinoids reduce calcium influx at presynaptic terminals of excitatory neurons. This action diminishes the release of neurotransmitters such as glutamate, SP, CGRP and BDNF,^62^ thereby attenuating excitatory drive within pain pathways.

Since the α2δ‐1 subunit exerts a regulatory influence on NMDARs and AMPARs in excitatory neurons,^63^ gabapentinoids therapeutic effects largely derives from their ability to inhibit the α2δ‐1‐linked NMDA receptors and Ca^2+^‐permeable AMPA receptorsactivated in pathological conditions. Gabapentinoids selectively target α_2_δ‐1–linked NMDA receptor complexes, sparing physiological NMDA receptors that lack this association. This receptor‐specific action accounts for their clinical utility against hypersensitivity while offering little benefit in nociceptive pain (they have not analgesic effect) as well as minimizes adverse effects typically observed with NMDA receptorantagonists.^63^

Compared with gabapentin, pregabalin shows stronger affinity for the α_2_δ subunit, translating into approximately 2.5 times greater potency. It is also absorbed more efficiently, reaching maximum plasma concentrations within about 1 h, whereas gabapentin requires 2–4 h to achieve peak levels.^64^

Gabapentin therapy is typically initiated at 300 mg/day, with gradual titration to 300 mg three times daily over the course of 3 days. Pregabalin, which is often easier to use in clinical practice, is usually started at 150 mg/day in three divided doses and can be increased to 300 mg/day within a week.^64^

Because the primary sites of action of gabapentinoids are intracellular, active transport into neurons is required. This uptake occurs mainly through system L‐amino acid transporters. Because these transporters are saturable, gabapentin absorption becomes dose‐dependent, with bioavailability decreasing significantly at higher doses. For example, the bioavailability of gabapentin is close to 80% at a 100‐mg dose but declines to roughly 27% when the dose is increased to 1600 mg. This dose‐dependent absorption helps explain its broad therapeutic window. Pregabalin, on the other hand, is transported by additional carriers and therefore displays linear, nonsaturable pharmacokinetics, maintaining a bioavailability greater than 90% across all dose ranges.^65^ Pregabalin demonstrated a more predictable dose–response relationship compared with gabapentin enabling faster titration and a simpler administration protocol. Pregabalin undergoes minimal metabolism and is primarily excreted unchanged in the urine; gabapentin requires more careful dosage adjustments in patients with impaired renal function^66^, ^67^, ^68^; pregabalin dose adjustment should be considered for patients with CLcr < 60 mL/min.^69^ To note, pregabalin offers positive effects in the short‐term treatment of generalized anxiety disorder (GAD),^70^ it appears to be efficacious in GAD‐dependent sleep disturbance.^71^ The most commonly reported adverse effects of gabapentinoids involve the nervous system or psychiatric domain, including dizziness, incoordination, abnormal gait, ataxia, peripheral oedema, weight gain and euphoria. The increasing use of these agents has also raised concerns regarding their potential for abuse. Nevertheless, current evidence does not provide strong or consistent support for addictive properties in patients without a prior history of substance misuse.^72^

## 4 DULOXETINE

Duloxetine belongs to a class of medications called serotonin (5‐HT) and NA reuptake inhibitors (SNRIs). These medications, primarily used to treat major depressive disorder, increase 5‐HT and NA levels in the neuronal synapse by blocking their subsequent reuptake. SNRIs are also the first FDA‐approved drug class for treating pain induce by diabetic neuropathy. Duloxetine is the only treatment recommended by the American Society of Clinical Oncology for chemotherapy‐induced neuropathy.^73^, ^74^

Unlike the antidepressant effects of duloxetine, which typically emerge after 2–4 weeks of treatment, its analgesic benefits in chronic pain can be observed within just a few days to one week^42^, ^75^ indicating a mechanism closely tied to the pathological condition. Duloxetine exerts its action by inhibiting central NA transporters, thereby increasing synaptic concentrations of this neurotransmitter. At the spinal level, duloxetine‐induced NA increase reduces hypersensitivity through α_2_‐adrenergic receptor activation, while at supraspinal sites enhanced noradrenergic tone acts on the LC to restore the function of compromised descending inhibitory pathways. Intraperitoneal administration of duloxetine has been shown to elevate spinal NA and induce analgesia even in late‐phase neuropathic pain models, although this effect diminishes over time.^76^, ^77^

Mechanistically,^54^ identified two distinct pathways through which duloxetine (and amitryptyline) alleviates neuropathic allodynia in animal models. The first operates early within the central nervous system and relies on descending noradrenergic inhibitory circuits, recruitment of α_2_‐adrenergic receptors, and the engagement of μ‐ and δ‐opioid receptors. The second unfolds more gradually, in the periphery, requiringα_2_‐adrenergic receptors other than δ‐opioid receptorS. Transcriptomic profiling of DRG further indicates that the peripheral mechanism is also linked to the suppression of neuroimmune responses triggered by nerve injury.

Recently, Arai et al.^78^ showed in a rat model of chronic neuropathic pain that duloxetine reduces abnormal LC activity, thus restoring endogenous analgesia. Nevertheless, in the chronic phase of neuropathic pain, the acute enhancement of pain threshold evoked by duloxetine, is largely sustained by spinal NA. This evidence justifies the drug efficacy despite some supraspinally contrasting role of NA (e.g., NA increase enhances pain in mPFC).

In patients with neuropathic pain, duloxetine is commonly prescribed at a daily dose of 60 mg.^79^ Following administration, peak plasma concentrations are reached after about 6 h, ranging from ~47 ng/mL with 40 mg twice‐daily dosing to ~110 ng/mL with 80 mg twice‐daily dosing. The drug has an elimination half‐life of approximately 10–12 h and a large volume of distribution, estimated at around 1640 L.^80^

Duloxetine is generally well tolerated, with adverse effects typically being mild. The most frequently reported are nausea, disturbances in sleep, constipation, sexual dysfunction and increases in heart rate and blood pressure. An additional concern is the elevated risk of bleeding in patients who are concurrently receiving anticoagulant therapy.^73^, ^74^, ^81^

## 5 GABAPENTINOIDS AND DULOXETINE IN THE CLINICAL PRACTICE

Through the years, gabapentinoids and duloxetine in addition to their primary indications have been approved for the clinical treatment of several painful conditions that strongly affect the quality of life of patients. The main conditions are the painful diabetic peripheral neuropathy (DPN), a common complication of diabetes mellitus, the postherpetic neuralgia (PHN), defined as a skin pain of long duration (≥3 months) after the acute Herpes zoster rash crusting, and fibromyalgia, a complex pain condition characterized by several symptoms including chronic widespread pain that is the most affecting the quality of life. Although these drugs only exert a symptomatic effect, without modifying the evolution of the related diseases, they show efficacy in reducing pain in these settings.

### 5.1 Gabapentinoids and duloxetine as single agents in pathological pain from different origin

#### 5.1.1 Randomized trials

Overall, gabapentinoids and duloxetine represent the most recommended drugs by current clinical practice guidelines provided by national and international scientific societies and associations involved in diabetes when a pharmacological treatment is needed.^82^ In fact,pregabalin and duloxetine, for instance, are recommended as first‐line agents for DPN. Concerning fibromyalgia, the quality of evidence of these drugs (especially duloxetine) in reducing pain is not high.^83^, ^84^ However, when the pharmacology therapy is needed, gabapentinoids (pregabalin, gabapentin and mirogabalin) and duloxetine are widely used.^84^

##### Pregabalin

The efficacy of pregabalin monotherapy in DPN was mainly established by a double‐blind, placebo‐controlled, multicentre trial including 146 patients randomized to receive pregabalin 300 mg/day (76 patients) or placebo (70 patients) for 8 weeks. The primary outcome measure, i.e. the mean pain score from daily patient diaries, was fully satisfied with pregabalin producing significant improvements *vs*. placebo (38% *vs*. 13% decrease in pain scores, respectively, *p* < .0001).^85^

Fundamental studies to establish the efficacy of *pregabalin in fibromyalgia* were performed by Arnold et al.^86^ and Crofford et al.^87^

The first was a randomized, double‐blind, placebo‐controlled, multicentre trial in which 750 patients were assigned to receive placebo of pregabalin (300, 450 and 600 mg/day) administered BID for 14 weeks.^86^ The primary outcome measure was the comparison of endpoint mean pain scores between each of the pregabalin groups and the placebo group. Compared with placebo‐treated patients, mean changes in pain scores at the endpoint in pregabalin‐treated patients were significantly greater (*p* < .001: 300 mg/day, −0.71; 450 mg/day, −0.98; 600 mg/day, −1.00).

The second study was a multicentre, double‐blind, placebo‐controlled, randomized withdrawal trial assessing the durability of pregabalin monotherapy effect in fibromyalgia.^87^ The trial included a 6‐week open‐label pregabalin‐treatment period with flexible titration to 300–600 mg/day. Patients who achieved at least a 50% of reduction in pain were randomized to a 6‐month double‐blind treatment with placebo or pregabalin. Overall, 63% of 1051 enrolled patients entered the double‐blind phase (*n* = 566). Double‐blind treatment was with placebo (*n* = 287) or the patient's optimal fixed dosage of pregabalin (*n* = 279). The primary outcome was time to loss of therapeutic response (LTR) (i.e., <30% reduction in pain from open label baseline) or worsening of fibromyalgia. Time to LTR was significantly longer for pregabalin *vs*. placebo. By Week 26, about 61% of pregabalin‐treated patients were responders compared with 32% of those on placebo (*p* < .0001).

The approval of *pregabalin in PHN* is also based on pivotal randomized trials.

Dworkin et al.^88^ conducted a multicentre, parallel‐group, double‐blind, placebo‐controlled, 8 weeks, randomized trial to assess the efficacy and tolerability of pregabalin in patients with PHN. The study enrolled 173 patients. Patients randomized to pregabalin (*n* = 89) started with a 3‐day 150 mg/day dose (50 mg TID) that was increased at 300 mg/day in the remaining days of the week. Starting from the second week, patients with creatinine clearance >60 mL/min received 600 mg/day (200 mg TID), the other remained on 300 mg/day (100 mg TID). The primary outcome was change in mean daily pain (11‐point NRS) from baseline to the endpoint. Both pregabalin doses achieved statistically significant reduction in endpoint main pain vs placebo with onset by Week 1 and persistence through the following weeks. Patients with ≥30% and ≥50% reduction in mean pain scores were in fact 63% *vs*. 25% and 50% *vs*. 20%, respectively (*p* < .001 in both cases). Adverse events reflected the established profile of pregabalin (e.g., dizziness, somnolence and dry mouth) and led to a relevant percentage of withdrawn (31.5%).

Some years later,^89^ published a multicentre, randomized, double‐blind, placebo‐controlled trial to assess the efficacy and tolerability of pregabalin in patients with PHN. The study enrolled 370 patients and treatment lasted 13 weeks. The study design was different from that of Dworkin et al.^88^ because three different pregabalin randomized groups *vs*. placebo were planned and the administration was BID and not TID as in Dworkin et al.^88^ However, even in this study, the dose administration according to the creatinine clearance was planned. Thus, 87 patients entered the 150‐mg/day group, 98 patients the 300‐mg day group and 90 patients the 300/600 mg/day. The placebo group consisted in 93 patients. The primary outcome was change from baseline to the endpoint in mean daily pain (11‐point NRS). This study confirmed that all pregabalin doses achieved statistically significant reduction in mean pain scores compared with placebo, with evident benefit from the first week. The safety profile was consistent with earlier pregabalin trials. Patients with reduced renal function experienced higher rates of discontinuation due to adverse events.

##### Gabapentin

Registrative studies for the use of *gabapentin in PHN* are represented by two main studies.^90^, ^91^

Rowbotham et al.^90^ conducted the first randomized, double‐blind, controlled trial in this field. Overall, 229 patients with PHN, were randomized to receive either gabapentin (*n* = 113) or placebo (*n* = 116) for 8 weeks. Gabapentin was titrated over the first 3 weeks from 300 mg/day to a maximum dose of 3600 mg/day (1200 mg TID). The primary outcome was the change in the average daily pain score (0–11 points, Likert scale). By intent‐to‐treat analysis, benefit of gabapentin at the end of Week 8 was significantly higher compared with placebo (average daily pain score from 6.3. to 4.2 and 6.5 to 6.9, respectively, *p* < .001).

The second study was performed by the Postherpetic Neuralgia Study Group including investigators from United Kingdom and Republic of Ireland.^91^ This multicentre double blind, randomized, placebo controlled 7‐week study was designed to further define the efficacy and tolerability of gabapentin in this patient's condition. The study enrolled a total of 334 patients. Gabapentin was titrated upward, initially from 300 to 1200 mg/day, and subsequently randomized up to 1800 mg/day (*n* = 115) or 2400 mg/day (*n* = 108). One hundred eleven patients entered the placebo (*n* = 111). The primary outcome was the change in average daily pain diary score (baseline week *vs*. last week, 0–11 points, Likert scale). Both pregabalin doses produced significantly greater reductions in mean pain scores: final difference *vs*. baseline was −34.5% for the 1800 mg dose, −34.4% for the 2400 mg dose compared with −15.7% for the placebo group (*p* < .001 in both cases). Gabapentin was substantially well tolerated and the most common adverse events (e.g., dizziness and somnolence) mainly developed during the titration phase.

##### Mirogabalin

From the clinical point of view, mirogabalin, currently only approved in Japan, differs from the other gabapentinoids because it has been developed with the treatment of peripheral neuropathic pain as the primary aim. Despite the conduction of several randomized clinical trials, a clear advantage of mirogabalin on pregabalin and gabapentin, has still not recognized by Western drug agencies. In the treatment of DPN, mirogabalin was shown to be effective in reducing the visual analogue pain score (VAS) compared with placebo. Mean change in the least square of the average daily pain score from baseline were −1.32 for placebo, −1.34 for 15 mg/day mirogabalin, −1.47 for 20 mg/day mirogabalin and −1.81 for 30 mg/day mirogabalin (*p* = .0027).^92^ When compared with placebo or pregabalin for DPN mirogabalin showed an advantage in term of at equipotent doses least square mean average daily pain score change from baseline *vs*. placebo were −0.22, −0.53, −0.94, −0.88 and −1.01 for the mirogabalin 5‐, 10‐, 15‐, 20‐ and 30‐mg/day arms, respectively, and −0.05 in the pregabalin arm (*p* < .05 *vs*. placebo for mirogabalin 15, 20 and 30 mg/day).^93^

Mirogabalin has also been tested in patients with PHN and efficacy results in comparison with placebo indicate a higher efficacy of the drug (15‐mg mirogabalin, −0.41; 20‐mg mirogabalin, −0.47; 30‐mg mirogabalin, −0.77).^94^

Finally, mirogabalin compared with placebo failed to satisfy the primary outcome (i.e., change in weekly average daily worst pain score at Week 13) in three multicentre randomized trials including patients with fibromyalgia.^95^

##### Duloxetine

In the same years, results from two pivotal trials investigating the efficacy of duloxetine monotherapy *vs*. placebo in *DPN* according to a double‐blind, randomized, multicentre trial design, were published.^96^, ^97^ In both studies the primary outcome measure, that is, the weekly mean score of 24‐h average pain severity (11‐point Likert scale) was satisfied. The study of Raskin et al.^96^ included 348 patients who received duloxetine 60 mg/day (QD) (*n* = 116) or 60 mg BID (*n* = 116) or placebo (*n* = 116) for 12 weeks. Patients in both duloxetine arms improved significantly (mean pain reductions of 64% to 68% *vs*. 43% in the placebo arm, *p* < 0.001) on the 24‐h average pain score.

The study of Goldstein et al.^97^ included 457 patients who received duloxetine 20 mg/day (QD), 60 mg/day (QD), 120 mg/day (60 mg BID) or placebo. Duloxetine arms at 60 and 120 mg/day showed statistically significant improvement (48% and 54% for 60‐mg group and 120‐mg group, respectively) compared with placebo (33%) on the 24‐h average pain score after 12 weeks of treatment.

Two main trials established the efficacy of *duloxetine in fibromyalgia*. Both studies were randomized, double‐blind, placebo‐controlled trials with the primary measure represented by the change in Brief Pain Inventory average pain severity score.^98^, ^99^ Although about 25% of patients was affected by major depressive disorder, the observed analgesic benefit was shown to be independent from the depressive status.

The efficacy of duloxetine in fibromyalgia was first shown in the trial of Arnold et al. that was 12 weeks in duration and enrolled only female.^98^ A total of 354 patients were assigned to receive duloxetine 60 mg QD, 60 mg BIT or placebo. Both duloxetine regimens produced significant reduction in pain compared with placebo (duloxetine 60 mg QD: 55%; *p* < .001; duloxetine 60 mg BID: 54%; *p* = .002; placebo: 33%), thus satisfying the primary endpoint. However, the 60‐mg BID regimen did not yield superior analgesic benefit compared with the 60‐mg QD dose, while it was associated with a greater frequency of adverse events and treatment discontinuation.

The second trial extended the evaluation to female and male as well as the duration to 6 months.^99^ A total of 520 participants were randomized to duloxetine 20 mg, 60 mg, 120 mg once daily or to placebo. As in the previous trial, duloxetine 60 mg/day and120 mg/day, both demonstrated a statistically superiority over placebo, whereas the 20 mg/day dose was ineffective. However, even in this case, dose escalation to 120 mg/day did not translate into a higher analgesia compared with the 60 mg/day regimen and was associated to a higher burden of side effects.

#### 5.1.2 Meta‐analysis including gabapentinoids and duloxetine as single agents

Overall, the efficacy of gabapentinoids and duloxetine in reducing pain in such diseases has also been substantially confirmed by systematic reviews and meta‐analysis published through the years.

##### Diabetic peripheral neuralgia

As far as *DPN* is concerned, a meta‐analysis consisting in 37 double‐blind, placebo‐controlled randomized trials, evaluated eight drugs, including gabapentin, pregabalin, mirogabalin and duloxetine.^100^ Both pregabalin and duloxetine were consistently superior to placebo across the established pain outcomes (primary outcome: continuous pain intensity). Mirogabalin was more effective than placebo whereas gabapentin showed no significant effect. The authors although described some limitations of their analysis, for instance short treatment durations (mostly ≤3 months), concluded that pregabalin and duloxetine are effective options in reducing pain from diabetes neuropathy despite the occurrence of significant adverse events.

A more recent Bayesian network meta‐analysis in DPN^101^ limited the comparison to the efficacy and safety of randomized controlled trials performed with only four agents, that is, pregabalin, duloxetine, gabapentin and oxcarbazepine, a keto‐analogue of carbamazepine. This last is not currently approved for the treatment of pain. All the active treatments were superior to placebo for reducing neuropathic pain. When drugs were directly compared for efficacy, gabapentin was the most effective followed by oxcarbazepine, duloxetine and pregabalin. When drugs were directly compared for withdrawal due to adverse events, gabapentin was the safest followed by pregabalin, duloxetine and oxcarbazepine. When indirect comparison was performed for the efficacy evaluation, duloxetine efficacy was significantly higher than that of pregabalin or oxcarbazepine. Finally, indirect comparison on patient withdrawal due to adverse events highlighted a safer profile for pregabalin, duloxetine and gabapentin.

##### Fibromyalgia

The first meta‐analysis that evaluated the pharmacological treatment of *fibromyalgia* ^102^ included 21 trials up to 2010 and compared eight different drugs in monotherapy and combination therapies (i.e., NSAIDs and sedative hypnotics) in patients with fibromyalgia. Results consistently showed the superiority of pregabalin, duloxetine and of further SNRIs, that is, milnacipran. Pregabalin was mainly associated with significant reductions in pain, improvements in sleep and fatigue; duloxetine showed meaningful effects on pain, mood and quality of life. Doses of pregabalin and duloxetine able to reduce pain on the 11‐point numerical rating scale (NRS) ranged from 300 to 600 mg and from 20 to 120 mg, respectively.

The last available publication^103^ is an overview of Cochrane systematic reviews based on meta‐analyses of randomized controlled trials of approved pharmacological treatments for fibromyalgia, including pregabalin and duloxetine. Together with milnacipran, duloxetine and pregabalin across more than 30 placebo‐controlled trials, emerged as the most consistently supported options, showing superiority over placebo for pain relief and functional outcomes. However, the effect sizes were consistently modest: only about 1 patient in 10 achieved substantial benefit, that is, at least a 50% reduction in pain, over 4–12 weeks of treatment. Despite this aspect, the authors concluded that pregabalin and duloxetine, together with milnacipran are the evidence‐based pharmacological choices for fibromyalgia.

##### PHN

A meta‐analysis based on randomized clinical trials, has been conducted to evaluate the efficacy of oral treatments in *PHN*.^104^ A total of eight studies were included although they had a high or unclear risk of bias and performed with heterogenous regimens. The subgroup analysis of five studies showed that, gabapentin and pregabalin as well as divalproex sodium, improved short‐term pain intensity. A further subgroup analysis of five studies showed that the assumption of anticonvulsants was associated with a 2.5 times likelihood to have a 50% or more reduction in pain after treatment compared with placebo. Based on these results, the benefit of gabapentinoids in PHN, although present, is not supported by high‐quality evidence. However, the limited number of studies meta‐analysed does not allow to draw definitive conclusions.

#### 5.1.3 Indications and dosage

In the context of the above‐mentioned pain syndromes, FDA approved gabapentin for the PHN, pregabalin and duloxetine for both indications (i.e., DPN and fibromyalgia) while EMA approved duloxetine only for DPN and pregabalin with the generic indication of peripheral and central neuropathic pain treatment without specifying the origin of pain.

The initial dose of gabapentin in PHN is 300 mg/day that is increased to 900 mg/day within 3 days (300 mg TID). The dose can subsequently be titrated up as needed for pain relief to a dose of 1800 mg/day (600 mg TID).

The dosage of pregabalin approved by FDA for DPN treatment is 50 mg TID (150 mg/day) at initiation. Titration up to 100 mg TID (300 mg/day) is allowed.

The recommended dose of pregabalin for fibromyalgia is 300 to 450 mg/day. The starting dose is 75 mg BID (150 mg/day). It may be increased to 150 mg BID (300 mg/day) within 1 week based on efficacy and tolerability. A further increase up to 225 mg BID (450 mg/day) is allowed in patients who do not experience sufficient benefit.

Posology of pregabalin recommended by EMA for neuropathic pain consists in a starting dose of 150 mg/day given BID or TID. According to the patient response and tolerability, the dose may be increased to 300 mg per day after an interval of 3 to 7 days, and if needed, to a maximum dose of 600 mg/day after an additional 1‐week interval.

The starting and maximum dose of duloxetine approved for the treatment of DPN by FDA and EMA is 60 mg/day, once daily. However, due to the large inter‐individual variability of duloxetine plasma concentration, EMA affirms that some patients unresponsive to 60 mg/day may benefit from a higher dose.

The recommended dose for duloxetine in fibromyalgia is 60 mg, once daily with a starting dose of 30 mg once daily for 1 week and subsequent increase to 60 mg once daily although some patients respond to the starting dose.

#### 5.1.4 Adverse events

Both gabapentinoids and duloxetine show a not very safe toxicity profile at the doses commonly used. Most common adverse reactions of *gabapentin* are dizziness, somnolence and peripheral oedema. Those of *gabapentin* are mainly represented by dizziness, somnolence, dry mouth, oedema, blurred vision, weight gain and thinking abnormal (primarily difficulty with concentration/attention). *Mirogabalin* show a tolerability profile substantially similar to that of the other gabapentinoids, in particular somnolence, dizziness and oedema. Patients treated with *duloxetine* mainly experience nausea, dry mouth, somnolence, constipation, decreased appetite and hyperhidrosis.

### 5.2 Gabapentinoids and duloxetine combination therapies

Based on the above reported data, it is clear that the ideal pharmacological treatment for pain related to DPN and fibromyalgia does not exist. Painful symptoms are often not adequately controlled with pharmacological treatments based on single agents. Thus, the combination of two drugs—more rarely more than two—is quite commonly used, although the level of evidence is currently low. To date, a limited number of clinical trials investigating the combination pregabalin–duloxetine in painful conditions related to DPN and fibromyalgia has been performed and only few of them are randomized trials (Table 1). The following paragraph will discuss the available randomized trial in DPN, fibromyalgia and other painful conditions such as the PHN.

**TABLE 1:** Randomized clinical trials evaluating gabapentinoids–duloxetine combinations.

Reference	Condition	Study design	Treatment Arm 1	Dose and regimen (Arm 1)	No. pts (Arm 1)	Treatment Arm 2	Dose and regimen (Arm 2)	No. pts (Arm 2)	Primary outcome	Secondary outcomes	Primary outcome efficacy results	Secondary outcome efficacy results	Side effects
Tanenberg (2011)^105^	DPN	Multinational, phase 4, open‐label, R, non‐inferiority, 3‐arm trial	ARM 1: DLX monotherapy (switch from GBP)	GBP tapered down (to 300 mg/d) while initiating DLX 30 mg once daily for 1 wk, then DLX 60 mg once daily; total treatment duration 12 wks	138	ARM 2: PGB monotherapy (switch from GBP)	GBP tapered down (to 300 mg/d) while titrating PGB to 300 mg/d; total treatment duration 12 wks	134	Improvement in pain reduction between DLX and PGB (non‐inferiority). Change from baseline to wk 12 in wkly mean of daily 24‐h pain severity (pain diary; 0–10)	Other outcomes: pt‐reported pain/functional measures and safety	Estimated mean change at wk 12: −2.6 (DLX) *vs*. −2.1 (PGB); treatment difference 0.49 (95% CI [−0.05 to 1.04]); met non‐inferiority criterion	DLX also met non‐inferiority *vs*. DLX + GBP	TEAEs consistent with known profiles; nausea, insomnia, hyperhidrosis, decreased appetite and vomiting more often with DLX or DLX + GBP *vs*. PGB; weight decreased with DLX and DLX + GBP *vs*. PGB
			ARM 3: DLX + GBP (add‐on)	Continue pre‐study GBP (≥900 mg/d) and add DLX 30 mg once daily for 1 wk, then DLX 60 mg once daily; total treatment duration 12 wks	135				Change from baseline to wk 12 in wkly mean of daily 24‐h pain severity (pain diary; 0–10)	Improvement in pain reduction (non‐inferiority). Other outcomes: pt‐reported pain/functional measures and safety		Significant superiority of DLX *vs*. PGB in the mean daily pain diary ratings at wks 2, 3, 5 through 11; of DLX and DLX + GBP *vs*. PGB at wks 2 and 8; not at wk 12	PGB: peripheral oedema DLX + GBP: more autonomic AEs than PGB
Tesfaye (2013)^106^	DPN	Multinational, R, double‐blind, parallel‐group study with initial standard‐dose period^a^ followed by R intensive period in nonresponders (COMBO‐DN)	Comb therapy (DLX + PGB) in nonresponders to monotherapy (standard doses) [wks 8–16]	[*Initial 8 wks: DLX 60 mg/d or PGB 300 mg/d*].^a^ Wks 8–16: for nonresponders: DLX 60 mg/d + PGB 300 mg/d (added the alternate drug); total 16 wks from baseline	170 (completed 149)	High‐dose monotherapy (DLX or PGB) in nonresponders to monotherapy standard doses (maximum dose) [wks 8–16]	[*Initial 8 wks: DLX 60 mg/d or PGB 300 mg/d*].^a^ Intensive 8 wks (wk 8–16) for nonresponders: DLX 120 mg/d or PGB 600 mg/d (dose escalation); total 16 wks from baseline	173 (completed 152)	≥50% or 2‐point reduction in BPI‐MSF‐24 h average pain score	Responder rate at wk 16 (≥30% pain reduction). Other pt‐reported outcomes and safety	≥50% responders: 52.1% (comb) *vs*. 39.3% (high‐dose monotherapy); *p* = .068	≥30% responders: 61.8% (comb) *vs*. 55.8% (high‐dose monotherapy); *p* = .565	TEAE: dizziness and somnolence were most frequent; frequency higher with high‐dose PGB *vs*. comb; nausea more frequent with comb *vs*. high‐dose monotherapy
Tesfaye (2022)^107^	DPN	Multicentre, R, double‐blind, crossover trial with active washout (OPTION‐DM)	3 treatment pathways (within‐subject): AMIT‐PGB (A‐P); PGB‐AMIT (P‐A); DLX‐PGB (D‐P)	Each pathway: 2‐wk titration; monotherapy for 6 wks then (if NRS > 3) add second drug for 10 wks; titrated towards MTD (AMIT up to 75 mg/d; DLX up to 120 mg/d; PGB up to 600 mg/d; reduced dosing schedule if eGFR 30–59). 7‐d washout between pathways. Total trial duration ~50 wks across three pathways	130 (84 completing at least two pathways (primary analysis set)	Comparator: comparisons among: A‐P followed by D‐P followed by P‐A, A‐P followed by P‐A followed by D‐P, D‐P followed by A‐P followed by P‐A, D‐P followed by P‐A followed by A‐P, P‐A followed by D‐P followed by A‐P, and P‐A followed by A‐P followed by D‐P	Within‐subject comparison across pathways (same participants); primary endpoint compares 7‐d average NRS daily pain during final wk of each pathway; pairwise differences reported	As in column ‘Treatment Arm 1’	Difference in 7‐d average daily pain NRS during the final wk (wk 16) of each pathway	Quality of life, mood and sleep measures (e.g., SF‐36, HADS and Insomnia Severity Index);	All three pathways produced similar pain reduction; mean NRS decreased from 6.6 at baseline to 3.3 at wk 16; no statistically or clinically significant difference. Pairwise mean differences (98.3% CI): D‐P *vs*. A‐P −0.1 (−0.5 to 0.3); P‐A *vs*. A‐P −0.1 (−0.5 to 0.3); P‐A *vs*. D‐P 0.0 (−0.4 to 0.4)	Lower scores of D‐P pathway for role limitation due to physical health than both P‐A and A‐P at wk 6 but not at wk 16, and higher insomnia (ISI score) than A‐P at both wk 6 and 16	Serious AEs did not differ between pathways; TEAE discontinuations: 7% (A‐P), 10% (D‐P) and 11% (P‐A) during comb treatment
Saxena (2024)^108^	DPN	Prospective, R, double‐blind, controlled pilot trial	PGB + DLX (fixed‐dose comb)	PGB 75 mg + DLX 30 mg twice daily (pts < 80 kg body weight); total duration 4 wks; taper after completion per guideline	15	PGB monotherapy	PGB 75 mg twice daily (pts < 80 kg body weight); total duration 4 wks; taper after completion per guideline	15	Pain intensity NRS over 4 wks	BPI‐MSF pain score; PDQ; quality of life (SF‐12); rescue analgesia use	At wk 4: NRS 1.80 ± 1.37 (comb) *vs*. 3.33 ± 1.11 (monotherapy), *p* = .002	At wk 4: BPI‐MSF 1.87 *vs*. 3.33; *p* = .002. PDQ 12.27 *vs*. 15.27; *p* = .011. PPARγ fold change: 3.49 *vs*. 1.520; *p* < .001	Side effects similar between groups; somnolence observed in 6.7% in comb group; nausea/vomiting in 6.7% in both groups; mostly within first 2 wks
Gilron (2016)^109^	Fibromyalgia	R, double‐blind, 4‐period crossover trial; each treatment period 6 wks at maximally tolerated dose	4 treatment periods: PGB monotherapy; PBO, DLX monotherapy, comb PGB‐DLX	Flexible titration to maximally tolerated dose; dosage ceilings: PGB 450 mg/d; DLX 120 mg/d. Each period 6 wks with taper/washout between periods	41 (39 completed ≥2 treatments)	Comparator: comparisons among the treatments reported in the column ‘Treatment Arm 1’	As in column ‘Dose and regimen (Treatment Arm 1)’	As in column ‘Treatment Arm 1’	Average pain intensity over the past 24 h (0–10 NRS) rated each morning upon arising and averaged over 7 ds at MTD	Worst pain intensity over the past 24 h and average nocturnal pain intensity during sleeping hours both rated each morning upon arising and averaged over 7 ds at MTD. Global pain relief; others: e.g., McGill Pain Questionnaire; Fibromyalgia Impact Questionnaire	Average pain at MTD: PBO 5.1; PGB 5; DLX 4; comb 3.7. Pain with comb lower than PBO (*p* = .001) and PGB (*p* = 0.001)	Moderate‐or‐greater global pain relief: 68% (comb) *vs*. 39% (PGB). McGill Pain Questionnaire scores: 16.1, 13.5, 12.0 and 8.4, respectively, for PBO, PGB, DLX and comb (*p* < .05 for the comb *vs*. PBO, PGB and DLX). Fibromyalgia Impact Questionnaire: 42.9, 37.4, 36.0 and 29.8, respectively, for PBO, PGB, DLX and comb (*p* < .05 for the comb *vs*. PBO, PGB, and DLX)	Moderate–severe drowsiness more frequent with comb *vs*. PBO; overall tolerability broadly comparable *vs*. monotherapy; MTDs lower in comb than in monotherapy
Wang (2025)^110^	PHN	R, double‐blind, crossover clinical trial	DLX + PGB	DLX 30 mg once daily + PGB 150 mg twice daily; increase DLX q 2 wks based on pain assessment up to 90 mg/d. Treatment period 6 wks; 2‐wk single‐blind PBO washout between treatments; total study duration 16 wks	110 (105 completed)	AMIT + PGB	AMIT 25 mg once daily + PGB 150 mg twice daily; increase AMIT q 2 wks based on pain assessment up to 75 mg/d. Treatment period 6 wks; 2‐wk single‐blind PBO washout between treatments; total study duration 16 wks	110 (103 completed)	Pain intensity (0–10 NRS) over 16‐wk study period	PSQI; HAMD‐17; Global pain relief categories (good/moderate/mild)	Both combs reduced NRS (*p* < .001 within groups) with no significant difference between groups	PSQI and HAMD‐17 decreased and SF‐36 increased after treatment in both groups; no significant between‐group differences. Proportions reporting good/moderate/mild pain relief were similar between groups (*p* = .351)	AEs included somnolence, insomnia, dizziness, fatigue, dry mouth, nausea and diarrhoea. Dry mouth was more common with AMIT + PGB (*p* = .008). Two withdrawals due to intolerable Aes
Krishnaprasad (2023)^111^	Neuropathic pain (mixed aetiologies)	Phase 3, R, double‐blind, double‐dummy, parallel‐group non‐inferiority study	FDC: PGB + DLX (low dose)	PGB 50 mg + DLX 20 mg/d titrated to max 75 mg + 30 mg twice daily, respectively; treatment duration 7 wks followed by 1‐wk follow‐up	164 (148 completed)	PGB monotherapy (higher dose)	PGB 75 mg/d titrated to max 150 mg twice daily; treatment duration 7 wks followed by 1‐wk follow‐up	164 (150 completed)	Mean change in pain intensity (NRS) at end of wk 7	Pain NRS change at wks 2, 3, 5; proportion of responders at wk 7; proportion of pts with ≥50% improve‐ ment in pain NRS from baseline to wk 7; mean change in pain total score from baseline to end of the treatment; Percentage of pts who required rescue medication; PGI‐I score at wk 7; CGI‐I score at wk 7; incidence of TEAE	Wk 7 mean change: −4.49 FDC *vs*. −4.66 PGB: non‐inferiority of low‐dose FDC demonstrated	No statistical significant difference between the two treatments (*p* > .05 for all the planned secondary outcomes)	Incidence of dizziness and somnolence comparable; peripheral oedema more frequent with PGB. Two discontinuations due to TEAEs in PGB group, none in FDC group

*Note*: For more detailed information, refer to the text and to the original papers.

Abbreviations: AE, adverse effect; AMIT, amitriptyline; BPI‐MSF, Brief Pain Inventory‐Modified Short Form; CGI‐I, Clinical Global Impression of Improvement; CI, confidence interval; comb, combination; d, day; DPN, diabetic peripheral neuropathy; DLX, duloxetine; eGFR, estimated glomerular filtration rate; FDC, fixed‐dose Comb; GBP, gabapentin; HAMD‐17, Hamilton Depressive symptoms; MTD, maximum tolerated dose; NRS, numerical rating scale; pt/pts, patient/patients; PBO, placebo; PDQ, PainDETECT Questionnaire; PGB, pregabalin; PGI‐I, Pt Global Impression of Improvement; PSQI, Pittsburgh sleep quality Index; R, randomized; TEAE, treatment‐emergent adverse event; wk/weeks, week/weeks.

^a^ Initial monotherapy phase not described.

#### 5.2.1 Randomized trials

##### Diabetic peripheral neuralgia

The first important randomized trial introducing an arm with the combination of a gabapentinoid with duloxetine dates to 2011.^105^ Although the trial was designed to evaluate the non‐inferiority of duloxetine on pregabalin in *DPN*, patients were randomized 1:1:1, thus 135 out of 407 patients entered the combination arm consisting of gabapentin and duloxetine. The primary outcome was a noninferiority comparison between duloxetine and pregabalin on improvement in the weekly mean of the diary‐based daily pain score (0–10‐point scale) at endpoint. Duloxetine group (*n* = 138) received 60 mg/day, pregabalin group (*n* = 134) 300 mg/day and the combination group duloxetine 60 mg/day and gabapentin ≥900 mg/day. Results showed a significant superiority *vs*. pregabalin according to the primary outcome for duloxetine monotherapy and for duloxetine plus gabapentin combination therapy (*p* < .05 and *p* < .01 vs pregabalin, respectively).

To date, the largest trial evaluating the combination pregabalin–duloxetine in DPN remains the COMBO‐DN.^106^ It is a two‐phase, multinational, double‐blind, parallel‐group, randomized clinical trial that was designed to test in patients insufficiently relieved by standard doses of pregabalin or duloxetine, whether adding the alternate drug to each of them, increased analgesia. Patients were randomized in four groups and entered an initial 8‐week monotherapy phase with either duloxetine 60 mg/day (two groups) or pregabalin 300 mg/day (two groups). Responders (≥30% pain reduction) exited the study, whereas non responders proceeded to the 8‐week comparison phase, according to the subsequent treatment regimen planned for each of the four groups. In this phase, patients received a combination at standard doses (i.e., duloxetine 60 mg/day plus pregabalin 300 mg/day) or to high‐dose monotherapy (i.e., duloxetine 120 mg/day or pregabalin 600 mg/day) after a titration week. The primary outcome was assessed with the Brief Pain Inventory Modified Short Form (BPI‐MSF) 24‐h average pain change. Secondary endpoints included other BPI‐MSF items, the Clinical Global Impression of Improvement scores, the Patient Global Impression of Improvement scores, the Neuropathic Pain Symptom Inventory (NPSI) questionnaire total score, the total and anxiety and depression subscale scores of the Hospital Anxiety and Depression Scale (HADS), the change in BPI‐MSF 24‐h average pain during initial therapy period, comparing standard doses of duloxetine and pregabalin.

A total of 804 patients participated in the initial phase and 339 nonresponders in the comparison phase. The primary endpoint did not differ significantly between combination (mean change −2.35) and pooled high‐dose monotherapy (mean change −2.16, *p* = .370). However, secondary endpoints, that is, 50% response rates showed differences between combination (52.1%) and monotherapy (39.3%) with a trend in favour of the combination (*p* = .068); the difference in the HADS anxiety subscale was statistically significant (*p* = .049).

Overall, tolerability was acceptable across regimens, and the observed adverse events reflected known class effects. In particular, treatment‐emergent adverse events (e.g., dizziness on pregabalin and nausea on duloxetine) were more frequent during the initial phase than during combination or high‐dose therapy.

Interestingly, data from the COMBO‐DN trial have been used to perform an exploratory post hoc cluster analysis to identify and characterize potential subgroups through their scores in the NPSI items. Bouhassira et al.^112^ investigated whether baseline symptom profiles assessed with the NPSI could predict differential treatment response to duloxetine, pregabalin or their combination. The analysis included patients from both phases of the COMBO‐DN. Using cluster methods on baseline NPSI scores, patients were stratified into homogeneous symptom subgroups, reflecting predominance of burning/spontaneous pain, paroxysmal pain, evoked pain or deep pain dimension. The main outcome was pain reduction measured on the BPI average score, with responder analyses (≥50% reduction) performed within groups. Across the entire population, baseline NPSI domains showed heterogeneous severity, but no cluster was clearly associated with superior response to one treatment over another. Both duloxetine and pregabalin produced significant pain reductions compared with baseline, but the magnitude of benefit did not differ across NPSI‐defined phenotypes. The combination therapy yielded consistently larger mean reductions in BPI average pain across most symptom clusters in patients with moderate and mild pain compared with high‐dose monotherapy, although the between‐treatment differences did not reach statistical significance at the subgroup level. Patients with severe pain showed instead a trend in favour of high‐dose monotherapy.

In recent years, the same authors completed a further complex trial to better define the role of the pharmacological treatment in *DPN*.^107^ The Optimal Pathway for Treating Neuropathic Pain in Diabetes Mellitus (OPTION‐DM) is a multicentre, double‐blind, randomized, two‐phases crossover trial designed to compare the efficacy of sequential monotherapy and combination therapy strategies in patients with painful DPN. The trial was conducted across 13 UK secondary‐care centres and enrolled 130 patients who were randomized to one of three treatment pathways: amitriptyline supplemented with pregabalin (A‐P), pregabalin supplemented with amitriptyline (P‐A), or duloxetine supplemented with pregabalin (D‐P). Each pathway consisted of two consecutive 16‐week treatment periods: an initial 6‐week monotherapy phase with dose titration to the maximum tolerated dose (up to amitriptyline 75 mg daily; duloxetine 120 mg daily; pregabalin 600 mg daily or maximum 300 mg daily if estimated glomerular filtration rate [eGFR] 30–59 per 1.73 m^2^), followed by supplementation with the alternate agent for patients who continue to report inadequate pain relief (NRS > 3). Patients crossed over sequentially to all three pathways allowing within‐patient comparisons.

The primary outcome was the difference in 7‐day average daily pain during the final week of each pathway (NRS 0–10). Of the patients, 64.6% completed at least two pathways. The 7‐day average NRS at Week 16, main pain scores were similar in all three pathways: A‐P: 3.3, D‐P: 3.3, P‐A: 3.4 (mean difference −0.1, 98.3% confidence interval [CI] [−0.5 to 0.3], for D‐P *vs*. A‐P and for D‐P *vs*. A‐P, −0.0, 98.3% CI [−0.4 to 0.4] for P‐A *vs*. D‐P). Thus, the primary endpoint of superiority between combination sequences was not met. However, when patients shifted from monotherapy to combination therapy, mean pain scores fell further: the addition of a second drug reduced pain by an additional −1.0 points (98.3% CI [0.6 to 1.3], *p* < .0001) between weeks 6 and 16, compared with −0.2 points for those remaining on monotherapy (98.3% CI [0.1 to 0.5], *p* = .085). This demonstrated that combination therapy was more effective than continued monotherapy escalation in patients with residual pain. Responder analyses and secondary outcomes, including global impression of change (PGIC) and sleep interference scores, supported this additive benefit.

Safety and tolerability profiles were consistent with the known adverse events of each agent. Combination regimens were associated with more frequent adverse events: a significant increase in dizziness was observed in the P‐A pathway, nausea in the D‐P pathway and dry mouth in the A‐P pathway. However, most were mild to moderate.

The lack of a placebo group could represent a limitation of this study that, however, showed key information on this matter.

Overall, OPTION‐DM showed that although amitriptyline, duloxetine and pregabalin are equally effective as first‐line monotherapies, the addition of a second agent provides clinically meaningful further pain relief. Thus, these finding support a stepwise escalation to rational dual therapy such as duloxetine plus pregabalin or amitriptyline plus pregabalin in patients with painful DPN who do not achieve adequate analgesia with monotherapy.

Most recently, a small randomized, controlled, single‐centre, 4‐week trial comparing the combination pregabalin–duloxetine *vs*. pregabalin monotherapy, was conducted by Saxena et al.^108^ Duloxetine (60 mg/day) and pregabalin (150 mg/day) were administered as combination therapy and pregabalin (150 mg/day) as monotherapy. The trial assessed pain intensity and quality of life measures and included a longitudinal study on the expression of two genes, that is, *PPARγ* and *Akt* in blood samples in order to investigate the molecular basis of neuropathic pain. Both genes play, in fact, a role in insulin regulation, inflammation and cell growth and in the context of neuropathic pain associated with DPN. However, the modulation of these genes has not been previously studied in patients on treatment with pregabalin–duloxetine combination. The study enrolled 34 patients and 30 were eligible for evaluation. Overall, the drug combination obtained major reduction in NRS‐pain scores and improvements were reported in several aspects of patient's life, for example, neuropathic symptoms, sleep, SF‐12 questionnaire scores.

As far as the mRNA expression of *PPARγ* and *Akt* genes is concerned, *PPARγ* gene expression was found to be significantly upregulated (*p* < .05) at the end of the last week (Week 4) in both treatment groups when an intragroup comparison with baseline was performed. According to an intergroup comparison the *PPARγ* gene expression was found to be significantly upregulated in the drug combination group as compared with the monotherapy group. These preliminary data appear interesting but need further investigation in larger cohorts of DPN patients.

##### Fibromyalgia

To date, only one randomized trial investigating the combination pregabalin–duloxetine in *fibromyalgia* is available. Gilron et al.^109^ conducted a randomized, double‐blind, four‐period crossover trial to evaluate whether the pregabalin–duloxetine combination provides superior symptom relief compared with pregabalin or duloxetine monotherapy or to placebo. The study enrolled 41 patients, each of whom received maximally tolerated doses of pregabalin monotherapy, duloxetine monotherapy; pregabalin–duloxetine combination, placebo according to four sequential treatment periods of 6 weeks in randomized order with washout intervals. The primary endpoint was the mean daily pain (0–10 point scale). Both pregabalin and duloxetine monotherapies were superior to placebo, and the combination regimen yielded a further reduction in pain scores beyond either monotherapy. Although the improvement with combination compared with monotherapies did not always reach conventional statistical thresholds, responder analysis indicated a higher proportion of patients attaining clinical meaningful pain relief with combination therapy. Daily pain during placebo, pregabalin, duloxetine and combination was in fact 5.1, 5.0, 4.1 and 3.7, respectively (*p* < .05 combination *vs*. placebo, and pregabalin). Percentages of patients with moderate global pain relief were 18%, 39%, 42% and 68%, respectively (*p* < .05 combination *vs*. placebo, pregabalin and duloxetine). Secondary outcomes provided convergent evidence: global pain relief, measures of sleep quality and functional scales, all favoured the combination regimen, with some domains showing statistically significant superiority over monotherapy (e.g., SF‐36 scores: 50.2, 55.7, 56.0 and 61.2, respectively, *p* < .05 combination *vs*. placebo, pregabalin and duloxetine; Medical Outcomes Study Sleep Scale scores: 48.9, 35.2, 46.1 and 32.1, respectively, *p* < .05 for combination *vs*. placebo, and duloxetine). The tolerability profile was consistent with the known adverse events of both drugs; drowsiness and dizziness were more common with pregabalin‐containing regimes, whereas nausea was more common with duloxetine‐containing regimens. The frequency of moderate‐to‐severe adverse effects was higher with the combination. Overall, this crossover trial showed that pregabalin and duloxetine each confer analgesic benefit in fibromyalgia and their concurrent administration provides additive improvement in pain and patient‐reported outcome.

Interestingly, in order to further exploit the potential of the combination pregabalin–duloxetine in fibromyalgia, a recent randomized controlled 24‐week trial investigated the benefit added by palmitoylethanolamide and acetyl‐L‐carnitine in this setting.^113^ Both palmitoylethanolamide, an endogenous fatty acid amide, and acetyl‐L‐carnitine, an acetyl‐group donor, alone or in combination have been shown to exert neuroprotective effects, thus may confer benefit in terms of pain relief in pathological pain of different origin.^114^, ^115^ Patients were initially treated (3 months) with duloxetine plus pregabalin and subsequently were randomized to continue the same treatment (*n* = 68) or to add palmitoylethanolamide 600 mg BID + ALC 500 mg BID (*n* = 62) for further 3 months. The primary outcome was cumulative disease severity (Widespread Pain Index, WPI) evaluated every 2 weeks. Secondary outcomes were the fortnightly scores of the patient‐completed revised Fibromyalgia Impact Questionnaire (FIQR) and the modified Fibromyalgia Assessment Status (FASmod) questionnaire. At the end of the study period, patients who received the add‐on treatment with palmitoylethanolamide and acetyl‐L‐carnitine showed additional significant improvements in primary outcome as well as in secondary outcomes (i.e., WPI scores steadily decreased in supplemented patients [*p* = .048] who also showed better outcome in FIQR [*p* = .033] and FASmod scores (*p* = .017).^113^

##### PHN

*PHN* consists in a skin pain of long duration (about 3 months) after the acute Herpes zoster rash outbreak. It is the most common complication of Herpes zoster and strongly affects the patient's quality of life in terms of general activity, sleep, and emotions. Patients in this condition may also experience anxiety and depression.

Wang et al.^110^ designed a double‐blind, randomized, two‐period crossover trial to directly compare two commonly used combination therapies in PHN: duloxetine supplemented with pregabalin vs amitriptyline supplemented with pregabalin. Although pregabalin is often combined with either a SNRI or a tricyclic antidepressant in clinical practice, the efficacy and tolerability of such combination have not been established in a controlled clinical trial.

A total of 220 patients with persistent PHN were randomized to one of two treatment sequences: duloxetine (30–90 mg/day) plus pregabalin (titrated up to 300–450 mg/day) (*n* = 110) or amitriptyline (25–75 mg/day) plus pregabalin (up to 300–450 mg/day) (*n* = 110) followed by a crossover.

The treatment started with a low dose of duloxetine 30 mg, once a day; amitriptyline 25 mg once a day; pregabalin 150 mg, twice daily. The dose of duloxetine or amitriptyline were increased every 2 weeks based on pain assessment.

Results showed that both regimens produced significant pain relief compared with baseline, and mean reductions in pain scores were essentially equivalent between the two combinations. Responder analysis (≥50% pain reduction) confirmed similar proportions of patients achieving clinically meaningful analgesia with both combinations. Secondary outcomes, including sleep quality, patient global impression of change and functional measures, were likewise improved in both arms without significant differences.

Common adverse events in both arms were mainly represented by somnolence, fatigue and dry mouth, although developed with different frequency (i.e., somnolence, 17%; fatigue, 12%; dry mouth, 11% in the duloxetine and pregabalin group; dry mouth, 26%; somnolence, 15%, fatigue, 14% in the amitriptyline and pregabalin group.

Even in this study, the lack of a placebo arm may represent a limitation. However, based on the study results, the combination of duloxetine and pregabalin in the treatment of patients affected by PHN significantly reduces pain scores compared with baseline and improves sleep quality and depression status. Moreover, the incidence of adverse effect to duloxetine–pregabalin combination is lower compared with the amitriptyline‐pregabalin combination (e.g., dry mouth). Thus, the combination duloxetine–pregabalin is particularly useful for patients poorly tolerant the side effects of tricyclic‐associated anticholinergic effects.

A recent trial was performed by Krishnaprasad et al.^111^ in adults with moderate‐to‐severe neuropathic pain of different aetiology, mainly including DPN, lumbar radiculopathy, PHN and other peripheral neuropathies. This is multicentre, double blind, randomized, phase III non‐inferiority trial comparing, over a 7‐week treatment period, a low fixed‐dose combination of pregabalin and duloxetine with higher‐dose pregabalin monotherapy, to demonstrate the non‐inferior analgesic efficacy of the combination. The primary endpoint, represented by the mean change in pain intensity on the NRS scale was satisfied (i.e., −4.49 in the combination group and −4.66 in the pregabalin group, with both treatments showing highly significant within‐group improvements from baseline (*p* < .0001).

#### 5.2.2 Clinical findings supporting a mechanism‐oriented rationale

Although the available clinical studies were not specifically designed to validate mechanistic hypotheses through biomarkers or neurophysiological measures, some findings appear consistent with the proposed mechanism‐oriented rationale underlying duloxetine–gabapentinoid combination therapy. Beyond reductions in pain intensity scores, some studies reported improvements in dimensions closely related to central sensitization and dysfunctional descending modulation, including sleep disturbances, pain interference, mood symptoms and quality of life.^107^, ^109^, ^110^ In fibromyalgia patients, Gilron et al.^109^ observed improvements not only in pain intensity of the combination vs pregabalin but also functional improvements according to Fibromyalgia Impact Questionnaire and Short‐Form McGill Pain Questionnaire, as well as global pain relief scores, suggesting a broader effect on multidimensional symptom burden. Similarly, OPTION‐DM by evaluating multidimensional outcomes including quality of life, mood, sleep, pain interference and neuropathic symptom profiles within the tested sequential combination treatments in DPN, showed that combination treatment was generally well tolerated and provided additional pain reduction in patients with suboptimal response to monotherapy.^107^ Moreover, the trial COMBO‐DN performed in the same pain condition, showed numerically higher 50% responder rates with duloxetine–pregabalin combination therapy compared with high‐dose monotherapy, supporting the concept that combining agents targeting complementary mechanisms may provide additional benefit without simply relying on dose escalation.^106^ Consistent with this interpretation, Krishnaprasad et al.^111^ demonstrated in patients affected by neuropathic pain of different aetiology, that a low‐dose fixed‐dose pregabalin–duloxetine combination achieved analgesic efficacy comparable to higher‐dose pregabalin monotherapy with similar tolerability, supporting a potential dose‐sparing effect of mechanism‐based combination therapy. Finally, Saxena et al.^108^ reported improvements in diabetic polyneuropathy not only in pain scores but also in pain interference, neuropathic symptom profiles, quality‐of‐life measures and *PPARγ* fold change, although the small sample size and pilot design limit the interpretation of these findings. Although these observations do not constitute direct mechanistic proof, they provide indirect clinical support for a mechanism‐oriented therapeutic strategy.

#### 5.2.3 Chemotherapy‐induced peripheral neuropathy

A further field in which the combination of gabapentinoids and duloxetine has been investigated is the peripheral neuropathy due to anticancer drugs. However, the currently available evidence is extremely limited and mainly consists of isolated case reports. Thus, these observations should be regarded as hypothesis‐generating rather than as supportive clinical evidence.

Paclitaxel is an antimicrotubular drug belonging to the class of taxanes. It is a neurotoxic agent that may require interruption due to this side effect with a detrimental impact on the patient's clinical outcome. Takenaka et al.^116^ reported the case of a gastric cancer patient, a 68‐year‐old man, who underwent surgery and then received eight standard cycles of paclitaxel‐based chemotherapy. The onset of severe peripheral neuropathy during the chemotherapeutic treatment importantly affected his quality of life. Thus, the patients were treated for 2 months with pregabalin (150 mg) plus trazodone (25 mg), but due to the persistence of symptoms, duloxetine (20 mg) was added. When an improvement was noted, duloxetine was increased to 30 mg. Overall, from the baseline to 5 months, VAS initially reported as 75 mm, decreased to 5 mm, despite pregabalin was reduced to 50 mg for the post 2.5 months.

A further example is represented by a case report related to a 53‐year‐old breast cancer patient who received the antimicrotubular eribulin, belonging to halichondrins, as third‐line chemotherapy and in previous treatments other neurotoxic agents (e.g., docetaxel).^117^ The patient started with mirogabalin single‐agent treatment afterwards duloxetine was added. The initial benefit received by mirogabalin was improved by duloxetine. The initial dose of mirogabalin (5 mg TID) was increased up to 22.5 mg/day. Duloxetine 20 mg/day was subsequently increased up to 40 mg/day. Overall, the administration of this combination therapy was able to attenuate neuropathy during the eribulin treatment.

Overall, these preliminary reports may suggest a potential role for duloxetine–gabapentinoid combinations in selected chemotherapy‐induced peripheral neuropathy patients. However, adequately powered controlled studies are required before any clinical recommendation can be proposed.

#### 5.2.4 Gabapentinoids–duloxetine interactions

Well‐established pharmacokinetic data indicate that duloxetine and gabapentinoids differ substantially in their metabolism. Duloxetine is primarily metabolized by CYP1A2 and CYP2D6,^80^ whereas gabapentin and pregabalin undergo negligible hepatic metabolism, are predominantly eliminated unchanged by renal excretion, and do not meaningfully inhibit or induce major CYP isoenzymes.^118^ Thus, no clinically relevant cytochrome P450‐mediated pharmacokinetic interaction is expected between duloxetine and gabapentinoids. Instead, the main safety concerns of this combination are likely to be pharmacodynamic, due to the overlap of CNS adverse effects (e.g., dizziness, somnolence, sedation and psychomotor impairment). However, available clinical studies do not consistently show a clinically relevant increase in these events with combination therapy compared with monotherapy. In COMBO‐DN^106^ and OPTION‐DM,^107^ the tolerability of duloxetine–pregabalin combination was comparable to high‐dose monotherapy or alternative treatment sequences. However, additive CNS effects remain clinically plausible, and in the fibromyalgia crossover trial of Gilron et al.^109^ drowsiness and dizziness were more frequent during pregabalin–duloxetine combination than during monotherapy. Thus, careful dose titration and monitoring are advisable, especially in older or frail patients and in those receiving other CNS depressants.

## 6 CONCLUSIONS

Overall, the clinical trials that over the years evaluated and led to the approval of the discussed drugs in the treatment of pathological pain of different origin, represent the clinical foundation on which current management is based. However, it is well recognized that patients enrolled in clinical trials constitute highly selected populations, which may differ substantially from those routinely treated by clinicians in real‐world settings. As a result, particularly in these pathological conditions, clinical efficacy in daily practice may be less effective and adverse events may occur more frequently and/or with greater severity.

Despite these limitations, pregabalin represents an important therapeutic option and is recommended as first‐line or early‐line in PDN and fibromyalgia. Its favourable pharmacokinetic profile also contributes to a manageable clinical use by enabling rapid straightforward dose titration. It can be administered in patients with impaired renal function (with dose adjustment) and exhibits minimal interactions with major drug classes. Finally, pregabalin is effective in GAD as well as in related sleep disorders. Gabapentin is an established therapeutic option, together with pregabalin, in PHN. In this context, the development of mirogabalin has not provided significant advances, because its efficacy and safety profile appear substantially comparable with those of the other two gabapentinoids. Duloxetine demonstrates clinically meaningful efficacy in PDN and fibromyalgia, further it is the only recommended drug against chemotherapy‐induced neuropathy. Duloxetine benefits from rapid titration and can be conveniently administered once daily.

Based on this evidence, by leveraging complementary mechanisms that target the specific alterations associated with chronic pain, the combination of gabapentinoids with duloxetine may offer potential improvements not only in pain relief but also in non‐pain symptoms such as mood and sleep disorders. On the other hand, the primary objective of chronic pain management extends beyond hypersensitivity elimination to encompass improved daily functioning, reduced pain‐related disability, and enhanced of quality of life.

To date, several studies have evaluated duloxetine–gabapentinoid combination therapy, with many reporting improvements in pain control and related symptoms compared with single agents. However, the available evidence should be interpreted cautiously, because many studies are characterized by relatively small sample sizes, short follow‐up durations and heterogeneous study designs, including the absence of uniform double‐blinding procedures. Thus, although these findings support the potential clinical utility of this pharmacological association in selected patients, further large‐scale and long‐term randomized studies are needed to better define the optimal treatment regimens in terms of posology (doses and administration) to maximize efficacy and limit side effects.

### NOMENCLATURE OF TARGETS AND LIGANDS

Key protein targets and ligands in this article are hyperlinked to corresponding entries online (http://www.guidetopharmacology.org) and are permanently archived in the Concise Guide to PHARMACOLOGY 2021/22.^119^

## AUTHOR CONTRIBUTIONS

**Stefania Nobili:** Investigation; data curation; formal analysis; writing—original draft. **Maurizio Evangelista:** Conceptualization; writing—review and editing. **Elena Lucarini:** Data curation; formal analysis; writing—original draft. **Elena G. Giugliano:** Investigation; data curation. **Carla Ghelardini:** Conceptualization; supervision; funding acquisition; writing—review and editing. **Laura Micheli:** Conceptualization; methodology; supervision; writing—review and editing. **Lorenzo Di Cesare Mannelli:** Conceptualization; methodology; supervision; project administration; writing—review and editing.

## CONFLICT OF INTEREST STATEMENT

The authors declare that no conflict of interests exists.

## Data Availability

No new data were created or analysed in this study. Data sharing is not applicable to this article.
